# Biofilm Challenge: Lactic Acid Bacteria Isolated from Bovine Udders versus Staphylococci

**DOI:** 10.3390/foods8020079

**Published:** 2019-02-20

**Authors:** Jonathan K. Wallis, Volker Krömker, Jan-Hendrik Paduch

**Affiliations:** University of Applied Sciences and Arts Hannover, Faculty II, Department Bioprocess Engineering, Microbiology, Heisterbergallee 10A, D-30453 Hannover, Germany; jonathan-wallis@hotmail.com (J.K.W.); Jan-hendrik.paduch@hs-hannover.de (J.-H.P.)

**Keywords:** lactic acid bacteria, biofilm, probiotic potential, staphylococci, mastitis

## Abstract

Mastitis poses a considerable threat to productivity and to animal welfare on modern dairy farms. However, the common way of antibiotic treatment does not always lead to a cure. Unsuccessful cures can, among other reasons, occur due to biofilm formation of the causative agent. This has attracted interest from researchers to introduce promising alternative therapeutic approaches, such as the use of beneficial lactic acid bacteria (LAB). In fact, using LAB for treating mastitis probably requires the formation of a beneficial biofilm by the probiotic bacteria. The present study investigated the ability of five LAB strains, selected on the basis of results from previous studies, to remove and to replace pathogenic biofilms in vitro. For this purpose, *Staphylococcus (S.) aureus* ATCC 12,600 and two strains—*S. xylosus* (35/07) and *S. epidermidis* (575/08)—belonging to the group of coagulase negative staphylococci (CNS) were allowed to form biofilms in a 96-well plate. Subsequently, the LAB were added to the well. The biofilm challenge was evaluated by scraping off and suspending the biofilm cells, followed by a plate count of serial dilutions using selective media. All the LAB strains successfully removed the staphylococcal biofilms. However, only *Lactobacillus (L.) rhamnosus* ATCC 7469 and *L. plantarum* 2/37 formed biofilms of their own to replace the pathogenic ones.

## 1. Introduction

Bovine mastitis is among the most prevalent and costly diseases the dairy industry is facing today. It has a substantial economic impact as a result of reduced milk yield and poor milk quality, milk losses due to discarded milk after antibiotic treatment, and high costs for drugs and veterinary services [1]. Furthermore, the outcome of antibiotic therapy, which is the common way of treating mastitis, is not always satisfactory [2]. According to Anderl et al. [3], the effect of antimicrobials can be reduced by biofilm formation of the causative agent. Schönborn and Krömker [4] found *Staphylococcus aureus* form biofilms in infected udders. In vitro studies suggest that many more pathogens may cause biofilm-related mastitis [5]. Therefore, novel approaches for treating the disease are needed. Administering probiotic lactic acid bacteria (LAB) is one of the most interesting alternatives to antibiotic treatment and has already shown promising results in previous studies [6,7,8]. The Food and Agriculture Organization of the United Nations (FAO) defines probiotics as “live microorganisms that, when administered in adequate amounts, confer a health benefit on the host” [9]. Many LAB have been given the GRAS (generally recognized as being safe) status by the Food and Drug Administration (FDA) because they are traditionally used to produce certain foods [9]. Additionally, several members of this group are regarded as commensals of the udder [10] and are therefore presumably harmless to consumers and patients.

According to Frola et al. [11], probiotic bacteria are required to form a beneficial biofilm inside the udder, serving as a barrier against pathogens. The present study investigates the ability of five selected LAB strains to disrupt and replace staphylococcal biofilms with beneficial biofilms of their own in order to exert a probiotic effect.

## 2. Materials and Methods

### 2.1. Selection of the Strains

For this study, five LAB strains (Table 1) were selected from the strain collection of the Faculty II, Department for Bioprocess Engineering and Microbiology of the University of Applied Sciences and Arts, Hannover, Germany, according to their biofilm-forming ability and their antimicrobial properties. All the strains had previously shown an ability to inhibit the growth of certain mastitis-causing pathogens [12]. Furthermore, they were all capable of forming a biofilm with a higher-than-average biomass (optical density >0.21 at 570 nm after crystal violet staining) in a recent study [13].

Three staphylococci strains that had already been used in previous studies [12] were selected for the biofilm challenge (Table 1). We chose one *S. aureus* strain as this pathogen is still one of the most important mastitis-causing pathogens and is frequently associated with persistent infections in the udder [14]. The two remaining strains belonged to the coagulase negative staphylococci (CNS) group, a bacterial group of increasing importance in modern dairy herds despite effective mastitis management programs. CNS have been found to cause an increased somatic cell count in infected udder quarters while persisting in the udder for at least 10 months [15]. They are able to induce clinical mastitis in dairy cattle [15]. However, most of the infections caused by CNS remain subclinical [16]. The two CNS strains used in this study (*S. xylosus* (35/07 and *S. epidermidis* (575/08)) were isolated from the udders of cows with mastitis. *S. xylosus* and *S. epidermidis* were among the five most prevalent CNS species isolated from bovine udders in a previous study [17].

### 2.2. Biofilm Assay

In order to examine the ability of the five LAB strains to disrupt staphylococcal biofilms and to establish probiotic biofilms of their own instead, a method based on Guerrieri et al. [18] was used. First of all, the staphylococci were allowed to preform biofilms. Subsequently, the LAB were added to the staphylococcal biofilms in order to perform the biofilm challenge. The biofilm formation of both species was assessed at three different points in time while incubating the bacteria together.

#### 2.2.1. Preformation of Biofilms by Staphylococci

After transferring the bacteria from the frozen stock culture to the brain heart infusion broth (Carl Roth GmbH+Co. KG, Karlsruhe, Germany), three consecutive subcultures were made, each being incubated aerobically at 37 °C for 24 h. The optical density of the third subculture was then adjusted to 0.6 at 540 nm wavelength corresponding to 7 log_10_ cfu/mL, and inocula of 200 µL were transferred to the wells of polypropylene 96-well plates (Greiner Bio-One GmbH, Frickenhausen, Germany). Biofilms were grown aerobically at 37 °C for 168 h (seven days). After 72 h, 50% of the broth from each well was replaced by fresh medium. This was performed by removing 100 µL with a pipette. Afterward, the wells were refilled with 100 µL of fresh broth. Then, the plates were incubated for 48 h under the same conditions. Subsequently, 50% of the growth medium was again replaced with fresh brain heart infusion broth, and the 96-well plates were incubated for a further 48 h.

#### 2.2.2. Biofilm Challenge

For the biofilm challenge, LAB inocula were passaged three times, as previously described for the staphylococci. For growing LAB, Tween 80-depleted de Man, Rogosa and Sharpe MRS broth was used, as described by Leccese Terraf et al. [19].

The brain heart infusion broth from the preformed staphylococcal biofilms in the 96-well plates was completely removed with a pipette and replaced with either 200 µL of LAB inoculum or with fresh MRS broth. The wells with fresh MRS broth on preformed staphylococcal biofilms served as negative control. The wells in which LAB were added to the staphylococcal biofilms were the challenge wells. Additionally, for every LAB strain, one well without a preformed pathogenic biofilm was filled with 200 µL inoculum to serve as positive control, and wells without a preformed biofilm were filled with pure MRS broth. The plates were incubated aerobically at 37 °C for 168 h (seven days). Medium refreshment was performed after 72 h, 48 h thereafter, and a further 48 h, as previously described for preformation of the staphylococcal biofilms.

#### 2.2.3. Assessment of Biofilm Formation

Assessment of biofilm formation was carried out along with each medium refreshment for LAB and staphylococci. First, the medium from the wells was discarded and the wells were washed three times with 0.85 % NaCl (*w*/*v*). After that, a sterile cotton wool swab (MWE, Corsham, Wiltshire, UK) was used to scrape off the bacterial cells from the well by pressing the swab against the inner surface and the bottom of the well and rotating it clockwise five times and anti-clockwise a further five times. The cotton tip of the swab was then broken off and dropped into an Eppendorf tube (Eppendorf AG, Hamburg, Germany) containing 1 mL of sterile Ringer’s solution (Merck AG, Darmstadt, Germany). This Eppendorf tube was vortexed for 30 s to detach the bacterial cells from the swab. From this suspension, tenfold dilutions were made, and the cfu/mL were determined via plate count using selective media. To detect LAB, MRS agar (Carl Roth GmbH+Co. KG, Karlsruhe, Germany) with a pH value of 5.5 was used to rule out growth of the staphylococci on this medium. Baird Parker agar (Carl Roth GmbH+Co. KG) with 5 % egg yolk tellurite emulsion (Carl Roth GmbH+Co. KG) was used to detect *S. aureus*, and Chapman agar (Carl Roth GmbH+Co. KG) with 5 % egg yolk emulsion (Carl Roth GmbH+Co. KG) was used to detect CNS. Exclusive growth of the bacteria on their specific medium had been confirmed in advance of the assay by performing a plate count from pure overnight cultures.

The whole assay was performed in triplicate.

### 2.3. Statistical Analysis

Microbial counts (cfu/mL) were converted into logarithmic values. The statistical analysis was performed with IBM SPSS Statistics 24. In order to examine possible effects of the LAB on biofilm growth of staphylococci, the data were subjected to a linear mixed model. Results were regarded as significant when the *p*-value was below 0.05. The staphylococci species and the LAB strains as well as the incubation time served as independent variables. The staphylococci cfu/mL were the dependent variable.

## 3. Results

### 3.1. Biofilm Assay

#### Assessment of Biofilm Formation

**Figure 1:**
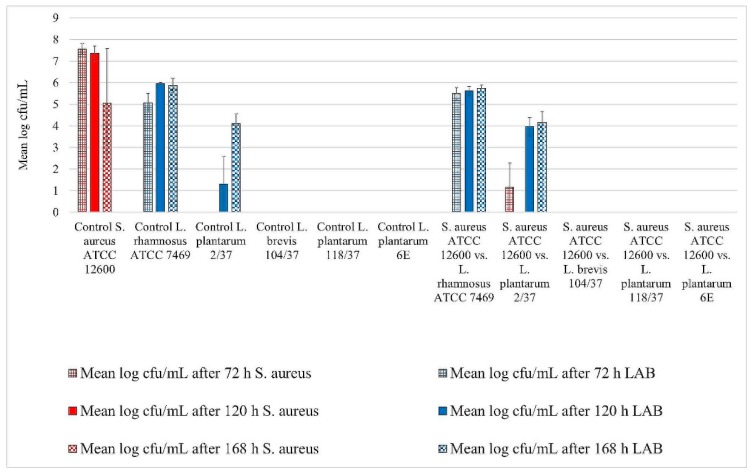
Biofilm challenge: *Staphylococcus aureus* ATCC 12,600 vs. lactic acid bacteria (LAB). Cfu/mL values are shown transformed by log (± standard error of the mean).

**Figure 2:**
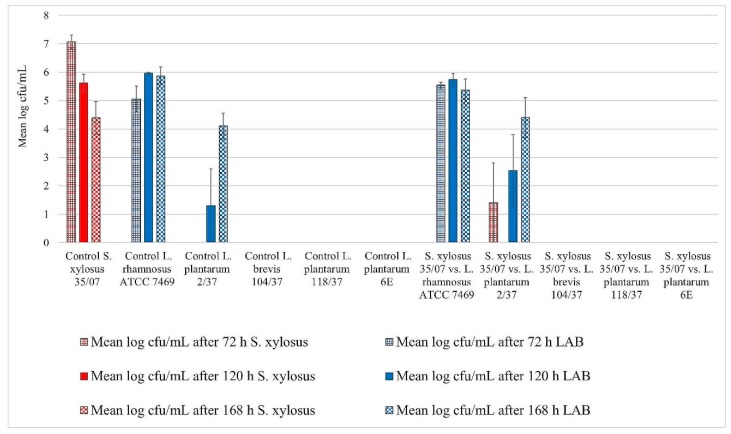
Biofilm challenge: *S. xylosus* (35/07) vs. LAB. Cfu/mL values are shown transformed by log (± standard error of the mean).

**Figure 3:**
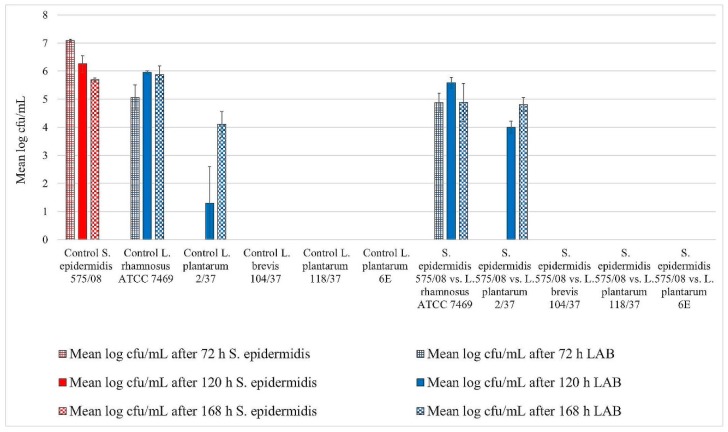
Biofilm challenge: *S. epidermidis* (575/08) vs. LAB. Cfu/mL values are shown transformed by log (± standard error of the mean).

All three staphylococci strains showed biofilm formation in the control well. Their biofilms remained detectable until the end of the trial (Figure 1, Figure 2 and Figure 3). The mean log cfu/mL values from the control wells seemed to decrease over time. *S. aureus* ATCC 12,600 revealed the highest mean cfu/mL values of the three staphylococci, increasing to approximately 7.6 log cfu/mL in the control after 72 h of incubation (Figure 1). In contrast, *S. xylosus* (35/07) showed the lowest staphylococcal cell count, achieving a mean log cfu/mL of approximately 4.4 in the control after 168 h of incubation (Figure 2). 

In the wells containing noninoculated MRS broth, we detected no bacteria throughout the trial. 

*L. rhamnosus* ATCC 7469 showed increasing cfu/mL values in the control wells, starting with approximately 5 log mean cfu/mL after 72 h of incubation. After 120 h of incubation, this strain revealed approximately 6 log mean cfu/mL, which remained constant until the end of the trial (Figure 1, Figure 2 and Figure 3). In the challenge wells containing *L. rhamnosus* ATCC 7469, this was the only detected strain. The mean log cfu/mL values from these wells were similar to those obtained from the control wells (Figure 1, Figure 2 and Figure 3). We found no biofilm formation by the three investigated staphylococci in the challenge wells after *L. rhamnosus* ATCC 7469 had been added (Figure 1, Figure 2 and Figure 3). 

However, we could still detect biofilm formation by *S. aureus* ATCC 12,600 and *S. xylosus* (35/07) in the challenge well despite the presence of *L. plantarum* 2/37 after 72 h of incubation during one of the three assay repetitions (Figure 1 and Figure 2). Biofilm formation by this strain was neither detected in the challenge nor in the control well after this time span. The first evidence of biofilm formation by *L. plantarum* 2/37 was found after 120 h of incubation in the control wells (approximately 1.3 log cfu/mL) as well as in the challenge wells, where we no longer found biofilms of the three tested pathogens (Figure 1, Figure 2 and Figure 3). The mean log cfu/mL values in the challenge wells after 120 h of incubation (2.5–4 log cfu/mL) were higher than the values obtained from the controls (Figure 1, Figure 2 and Figure 3). After 168 h of incubation, *L. plantarum* 2/37 maintained a biofilm in the challenge wells against all the three investigated staphylococci and was still present in the control wells, with the calculated values in both kinds of wells being more similar (4.1–4.8 mean log cfu/mL).

In the challenge wells containing the strains *L. brevis* 104/37, *L. plantarum* 118/37, and *L. plantarum* 6E, no staphylococcal biofilms were found after 72 h of incubation. However, none of them formed a detectable biofilm of their own either in the control or in the challenge well.

### 3.2. Statistical Analysis

The statistical analysis revealed a significant reduction in staphylococcal growth by LAB (*p* < 0.05). Furthermore, the incubation time significantly affected the reduction (*p* < 0.05). We observed no differences between the five investigated LAB strains.

## 4. Discussion

The method for evaluating biofilm formation applied in this study represents a culture-based approach involving specific growth media in order to differentiate between LAB and staphylococci. According to Jahid and Ha [20], culture-based methods are the most useful technique to differentiate known strains from mixed-species biofilms. The successful use of Baird Parker and MRS agars to distinguish *S. aureus* and LAB populations was already described by Gonzalez et al. [21]. The crystal violet assay is a common method used to assess biofilm formation. Crystal violet binds nonspecifically to viable and to dead bacterial cells as well as to matrix components [22]. Therefore, measuring the optical density after crystal violet staining is a valuable tool to establish the total biomass of a biofilm. However, it cannot distinguish between different species in a mixed-species biofilm. For this reason, crystal violet staining was not an option for evaluating the specific share in a biofilm of staphylococci and LAB. Nevertheless, the LAB strains included in this study were selected on the basis of the results of a crystal violet assay performed in a previous study [13], where a strong biofilm had formed on a polypropylene surface after 72 h incubation under the same conditions provided in this study. Therefore, we assume that it was due to the culture-based method for biofilm quantification that we found no biofilm formation by three of the LAB strains and not due to the growth conditions. Fernández Ramírez et al. [22] stated that results of a crystal violet assay might correlate poorly with those obtained by culture-based methods as not all the stained biomass in a mature biofilm has to consist of culturable bacterial cells. These findings could explain why we did not observe biofilm formation by *L. brevis* 104/37, *L. plantarum* 118/37, and *L. plantarum* 6E in the present study. Furthermore, Klinger-Strobel et al. [23] stated that loss of biomass could occur due to the washing step commonly performed in crystal violet assays. However, this might account even more for the culture-based technique applied in our study as the biofilms were washed three times using pipette suction in order to remove unbound cells prior to scraping off the biofilm by rotating a cotton swab in the well. The previously performed crystal violet assay involved only one washing step using gently flowing tap water.

We could not find any biofilm formation by staphylococci in the challenge well after LAB had been added to it, except for one repetition of the assay during which we detected *S. aureus* ATCC 12,600 and *S. xylosus* (35/07) biofilms in the first assessment of the challenge against *L. plantarum* 2/37. As we found no evidence of LAB and staphylococci being present in the same well at the same time, we can deduce that there was no formation of mixed-species biofilms containing both LAB and staphylococci. The staphylococci maintained a strong biofilm in the control well containing MRS broth where LAB were absent. Therefore, we assume that the LAB were responsible for eradicating the staphylococcal biofilm from the challenge well, and the effect was not due to the MRS broth. Furthermore, the statistical analysis revealed a significant growth reduction (*p* < 0.05).

*L. rhamnosus* ATCC 7469 appeared to be very effective at removing biofilms formed by staphylococci. It might be suitable for a probiotic remedy due to its high growth rates and its ability to form a strong biofilm after a short period of time. According to James et al. [24], high growth rates may lead to dominance over other biofilm formers when existing in the same habitat. Nonetheless, this strain showed a below-average adhesion to epithelial cells from the bovine udder in previous in vitro studies [13], which might interfere with the strain’s ability to form a beneficial biofilm in the udder under in vivo conditions. *L. plantarum* 2/37 seems to be a rather slow-growing strain. As adhesion to the epithelium and subsequent biofilm formation accounts for the ability of a potential probiotic strain to maintain its presence in the host and its positive effects over time [25], slow formation of a stable biofilm might be a disadvantage. However, *L. plantarum* 2/37 did finally form a stable biofilm and showed a strong adhesion ability to epithelial cells of the bovine udder during previous investigations [13]. Therefore, this strain might still be a potential candidate for a probiotic remedy. *L. brevis* 104/37, *L. plantarum* 118/37, and *L. plantarum* 6E revealed the ability to eradicate staphylococcal biofilms fast and effectively. Nonetheless, these strains were neither able to form a detectable biofilm of their own in the control nor in the challenge well. The three aforementioned strains showed a strong antimicrobial activity, which is in line with the results of Diepers et al. [12]. However, their inability to form a detectable biofilm of their own might interfere with their probiotic potential, as previously explained for *L. plantarum* 2/37. 

With regard to mastitis treatment based on LAB, further research is needed, including in vivo studies, as the bacteria might show a different behavior concerning biofilm formation in a milky environment [26]. Additionally, their safety for consumers and patients is yet to be verified, since mastitis by LAB as well as severe infections in humans are described in literature [9,27].

## 5. Conclusions

The present study focused on the ability of five LAB strains to disrupt and replace pathogenic biofilms formed by staphylococci with a presumably beneficial biofilm of their own in vitro. The results recommend two strains—*L. rhamnosus* ATCC 7469 and *L. plantarum* 2/37—for further investigations, focusing on their safety for consumers and patients as well as their beneficial properties on udder health under in vivo conditions.

## Figures and Tables

**Table 1:** Bacterial strains used in this study.

Strain	Origin
*L. rhamnosus* ATCC 7469	American Type Culture Collection
*L. plantarum* 2/37	Quarter milk samples with normal secretion (somatic cell count <100,000/mL, no pathogen detected)
*L. brevis* 104/37	
*L. plantarum* 118/37	
*L. plantarum* 6E	Bedding sample
*S. aureus* ATCC 12,600	American Type Culture Collection
*S. xylosus* (35/07)	Quarter milk sample from udders of infected cows
*S. epidermidis* (575/08)	
